# Plant Cell Wall Proteomes: The Core of Conserved Protein Families and the Case of Non-Canonical Proteins

**DOI:** 10.3390/ijms23084273

**Published:** 2022-04-12

**Authors:** Hélène San Clemente, Hasan Kolkas, Hervé Canut, Elisabeth Jamet

**Affiliations:** Laboratoire de Recherche en Sciences Végétales, Université de Toulouse, CNRS, UPS, Toulouse INP, 31320 Auzeville-Tolosane, France; sancle@lrsv.ups-tlse.fr (H.S.C.); hasan.kolkas@lrsv.ups-tlse.fr (H.K.); canut@lrsv.ups-tlse.fr (H.C.)

**Keywords:** cell wall, cell wall protein, early divergent plant, flowering plant, green lineage, proteomics

## Abstract

Plant cell wall proteins (CWPs) play critical roles during plant development and in response to stresses. Proteomics has revealed their great diversity. With nearly 1000 identified CWPs, the *Arabidopsis thaliana* cell wall proteome is the best described to date and it covers the main plant organs and cell suspension cultures. Other monocot and dicot plants have been studied as well as bryophytes, such as *Physcomitrella patens* and *Marchantia polymorpha*. Although these proteomes were obtained using various flowcharts, they can be searched for the presence of members of a given protein family. Thereby, a core cell wall proteome which does not pretend to be exhaustive, yet could be defined. It comprises: (i) glycoside hydrolases and pectin methyl esterases, (ii) class III peroxidases, (iii) Asp, Ser and Cys proteases, (iv) non-specific lipid transfer proteins, (v) fasciclin arabinogalactan proteins, (vi) purple acid phosphatases and (vii) thaumatins. All the conserved CWP families could represent a set of house-keeping CWPs critical for either the maintenance of the basic cell wall functions, allowing immediate response to environmental stresses or both. Besides, the presence of non-canonical proteins devoid of a predicted signal peptide in cell wall proteomes is discussed in relation to the possible existence of alternative secretion pathways.

## 1. Introduction

Plant cell walls are an important cell compartment playing critical roles in development as well as biotic and abiotic stresses. During cell growth, the so-called primary cell walls contain intricate networks of polysaccharides (90–95% of the total mass), cell wall proteins (CWPs) (5–10%), nutrient minerals in the apoplast, which can be defined as the soluble fraction of the extracellular matrix, as well as aromatic compounds in some plants, such as monocots and bryophytes [1]. At the end of growth, secondary walls can be synthesized. Covalent cross-linkings involving either hemicelluloses such as glucuronoarabinoxylans and lignin monomers, or structural proteins such as extensins reinforce the cell wall structure [2].

In primary walls, the main polysaccharides are pectins, hemicelluloses and cellulose. Pectin molecules are of three types [3]: (i) homogalacturonans (HGs), which are secreted as methylesterified molecules and can be demethylated *in muro* by pectin methylesterases (PMEs) to form the so-called egg box structures after ionic interaction with calcium ions [4]; type I rhamnogalacturonans (RGI); and type II rhamnogalacturonans (RGII), which form dimers with boron ions. Major hemicelluloses can be xyloglucans in dicot plants, glucuronoarabinoxylans in monocots or mannans in bryophytes [1,5,6]. Finally, cellulose is the main load-bearing polymer present in all cell walls. Cellulose molecules are the simplest polymers in cell walls. They are constituted of linear chains of (1- > 4)-β-D-glucose organized in microfibrils, which are synthesized by cellulose synthases at the plasma membrane [7].

The capacity of the cell wall to expand or to be modified relies on the activities of numerous CWPs. For example, the local interactions at the level of biomechanical hotspots between cellulose microfibrils and hemicelluloses, such as xyloglucans, can be modified by expansins, thus determining the loosening capacity of cell walls [8]. Class III peroxidases (CIII Prxs) can polymerize phenolic molecules, such as lignin monomers or tyrosine residues of structural proteins, such as extensins [9]. Besides, signaling molecules, such as peptides or oligogalacturonides, can be released from proteins or polysaccharides thanks to cell wall hydrolase activities [10,11]. These external signals are perceived by plasma membrane receptors which transmit the information to the inside of the cell, thus triggering regulatory mechanisms involved in development or in response to environmental cues. These few examples highlight some of the roles played by CWPs.

Proteins which were not predicted to be secreted were identified in all the cell wall proteomes characterized so far. They were named non-canonical CWPs and could have been considered as contaminant proteins [12,13]. Alternative secretory routes have been described in bacteria and mammals. They were grouped under the unconventional protein secretion (UPS) pathways. The proteins following these routes are leaderless and share particular features, such as amino acid content, secondary structure or disordered regions [14,15]. The question of the existence of such alternative secretion pathways in plants is still a matter of debate.

The diversity of CWPs were revealed since the 2000s with the development of dedicated cell wall proteomics studies [16]. These studies were boosted by the description of plant genomic sequences, starting with that of *Arabidopsis thaliana* [17], in parallel with the development of mass spectrometry (MS)-based identification of proteins [18]. Nowadays, the strategies for isolation of proteins from cell walls and their identification are well-established [16,19]. New cell wall proteomes are described, thus allowing drawing a general picture. The aim of this article is to (i) provide an update on plant cell wall proteomics, (ii) define a core cell wall proteome comprising the protein families which are conserved in 13 yet described cell wall proteomes of dicot and monocot plant species, and (iii) discuss the case of the non-canonical proteins devoid of a predicted signal peptide which have been identified in all the cell wall proteomes.

## 2. An Overview of the Selected Cell Wall Proteomes

For this analysis, we have selected proteomic studies from 13 plant species, corresponding to 36 independent studies (Table 1). For a given plant, the cell wall proteome, as considered in this article, encompasses all the CWPs identified at least once in at least one organ or in cell suspension cultures. Among the selected plants, there are one bryophyte (*Marchantia polymorpha*), eight dicots (*A. thaliana*, *Linum usitatissimum*, *Medicago sativa*, *Populus spp*, *Solanum lycopersicum*, *S. tuberosum*, *Gossypium hirsutum* and *Camellia sinensis*) and four monocots (*Saccharum officinarum*, *Triticum aestivum*, *Oryza sativa* and *Brachypodium distachyon*). Different organs have been analyzed (thallus, hypocotyls, root, stem, leaf, or fruit) as well as cell suspension cultures and their culture media. A few experiments deal with the exposure to environmental constraints, such as temperature stress [20,21,22], salicylic acid treatment [23], β-aminobutyric acid treatment [24], phosphate starvation [25] or pathogen infection [26]. All these proteomes were chosen because most of them have been obtained in similar experimental conditions (Section 3), they have a minimal size of 100 CWPs and the available data have allowed a new expert annotation of all the identified proteins and their sorting into CWPs or presumed intracellular contaminants (Section 3). All of them, except for *T. aestivum* [27], can be found in *WallProtDB-2* (https://www.polebio.lrsv.ups-tlse.fr/WallProtDB/) (accessed on 6 April 2022). [28]. The number of CWPs of the selected proteomes varies from 106 (*L. usitatissimum*) to 989 (*A. thaliana*) (Table 1).

## 3. How to Define CWPs and to Explore Cell Wall Proteomes?

The fact that cell walls are open compartments is a major difficulty for the preparation of cell wall fractions devoid of intracellular contaminants. From a historical point of view, two main strategies have been used: (i) the recovery of extracellular fluids after vacuum infiltration as a “non-destructive protocol” [33]; and the purification of cell walls followed by the elution of proteins with salt solutions, as a “destructive protocol” established for *A. thaliana* etiolated hypocotyls [30,31].

**Figure 1:**
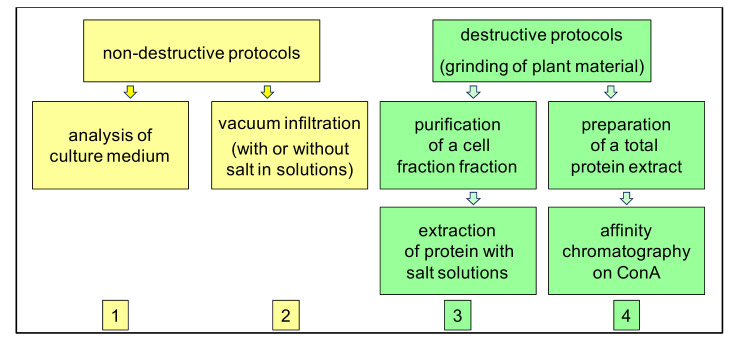
The four types of protocols (1–4) which have been used to study the extracellular proteome of plants. They can be qualified as non-destructive (1,2), or destructive (3,4), depending on whether they start with a grinding step or not.

Then four main strategies were used for different plant and various organs [16] (Figure 1): non-destructive protocols involving either (1) a vacuum-infiltration step of plant tissues or (2) the analysis of culture media; or destructive protocols starting with (3) the purification of a cell wall fraction, followed by extraction of the proteins with salt solutions or (4) the isolation of *N*-glycoproteins from a total protein extract through Concanavalin A (ConA) affinity chromatography. This latter strategy is based on the fact that extracellular proteins are routed through the secretory pathway where many of them become *N*-glycosylated [61]. All these approaches have proven to be complementary and their combination has allowed enlarging the coverage of cell wall proteomes [29,48]. The steps of protein separation or protein identification could also vary [16]. However, they tend to be more and more similar with the development of shotgun mass spectrometry (MS) analyses by LC-MS/MS [34]. Altogether, it is now reasonable to investigate the different proteomes in order to (i) define a core cell wall proteome and (ii) identify proteins possibly directed to the extracellular space through alternative secretion pathways.

The next step was to identify bona fide CWPs among the identified proteins. Indeed, the presence of proteins well-described as intracellular proteins, such as proteins participating in protein synthesis has been reported in nearly all the cell wall proteomics studies (Section 5).

The proteins present in the apoplast and in the cell wall are assumed to be secreted through the secretion pathway thanks to a signal peptide which targets them to the reticulum endoplasmic during their biosynthesis. Several bioinformatics programs can be used to predict which proteins could be found in the extracellular space, such as TargetP [62], SignalP [63], Phobius [64], Predotar [65] or LocTree3 [66]. Besides, it is possible to predict the presence of trans-membrane domains indicating a localization at the plasma membrane or an anchoring on its external side through a glycosylphosphatidylinositol (GPI)-anchor. Databases or bioinformatics programs, such as Aramemnon [67], TMPred [68], TMHMM [69], PredGPI [70] or GPI-SOM [71], can be used to this end. The ProtAnnDB annotation tool collects such predictions for 21 plant species [72].

Other proteins expected to be intracellular have also been identified in cell wall proteomes (Section 5). They could be considered as contaminant proteins or as non-canonical CWPs. However, one cannot exclude the existence of alternative routes of secretion which have been demonstrated in bacteria and in mammals for which dedicated software has been designed (Secretome P) [14].

In all the cell wall proteomes included in this study, we have chosen to consider proteins as CWPs if (i) a signal peptide could be predicted by at least two different bioinformatic programs, (ii) no ER retention signal could be predicted and (iii) less than two trans-membrane domains could be predicted, or if an experimental work already showed that proteins of the same family were located in the extracellular space. Note that signal peptides can be predicted as trans-membrane domains by some bioinformatic programs since they share common properties such as the presence of stretches of hydrophobic amino acid residues. We are thus left with three categories of CWPs: (i) those having a predicted signal peptide; (ii) those having both a predicted signal peptide and a GPI-anchor; and (iii) those which have experimentally been proven to be extracellular. In addition, we have considered proteins having an extracellular domain possibly interacting with ligands, such as peptides or oligosaccharides; a predicted trans-membrane domain, and a predicted kinase cytoplasmic domain. As receptor kinases, such proteins play critical roles in the transfer of information from the outside of the cell to its inside [73,74,75].

Since we want to analyze different cell wall proteomes, it is necessary to homogenize the functional annotation of the CWPs. This precaution will avoid relying on automatic annotations based on sequence comparisons which can be misleading. All the proteins selected as CWPs were re-annotated according to the presence of domains such as PROSITE [76], Pfam [77] or InterPro [78].

## 4. A Core Cell Wall Proteome: The Conserved CWPs Families and Their Possible Roles in Cell Walls

The systematic re-annotation of CWPs after the presence of functional domains has allowed grouping them into nine functional classes [12], which have been found in various proportions in the cell wall proteomes of the 13 studied plant species:

- Proteins acting on cell wall carbohydrates (PACs) belong to the major functional class in all the cell wall proteomes accounting for up to 25% of the CWPs. It comprises expansins [79] as well as glycosyl hydrolases (GHs), carbohydrate esterases (CEs) such as pectin methylesterases (PMEs) and polysaccharide lyases (PLs). The description of the latter protein families can be found in the Carbohydrate-Active enZYmes Database (CAZyDB, http://www.cazy.org) (accessed on 6 April 2022) [80].
- Oxido-reductases (ORs) include class III peroxidases (CIII Prxs), blue copper binding proteins, berberine bridge oxido-reductases, multicopper oxidases and laccases. The CIII Prxs and blue copper binding proteins are described in the Redoxibase (https://peroxibase.toulouse.inrae.fr) (accessed on 6 April 2022) [81] and the two latter protein families are included in CAZyDB.
- Proteases include several sub-families, such as Ser proteases (subtilisins), Ser carboxypeptidases, Cys proteases (papain family) and Asp proteases (e.g., pepsin family) [82,83].
- Proteins related to lipid metabolism comprise lipid transfer proteins (LTPs) [84], GDSL (Gly-Asp-Ser-Leu) lipases/acylhydrolases [85], glycerophosphodiester phosphodiesterases-like (GDPLs) [86] and phospholipases [87].
- Proteins possibly involved in signaling include arabinogalactan proteins (AGPs) [88], precursor of signaling peptides [89] and receptor kinases [73,74,75].
- Proteins with interaction domains comprise proteins interacting with other proteins, such as enzyme inhibitors, or with cell wall carbohydrates, such as lectins [73].
- Structural proteins, such as hydroxyproline-rich glycoproteins (HRGPs), are scarcely represented in cell wall proteins because many of them are covalently cross-linked in cell walls and thus difficult to extract. A study has particularly succeeded in the identification of several extensins, Pro-rich proteins and leucine-rich extensins by using a dedicated protocol including a trypsin digestion applied directly on cell walls [90].
- Miscellaneous proteins include proteins which cannot be classified into the other groups. Among others, they include dirigent proteins [91], purple acid phosphatases [92], phosphate-induced (phi) proteins (EXORDIUM-like proteins) [93] and germins [94].
- Proteins of unknown function can represent more than one tenth of the cell wall proteomes, suggesting new functions or new biological activities yet to be described.

**Figure 2:**
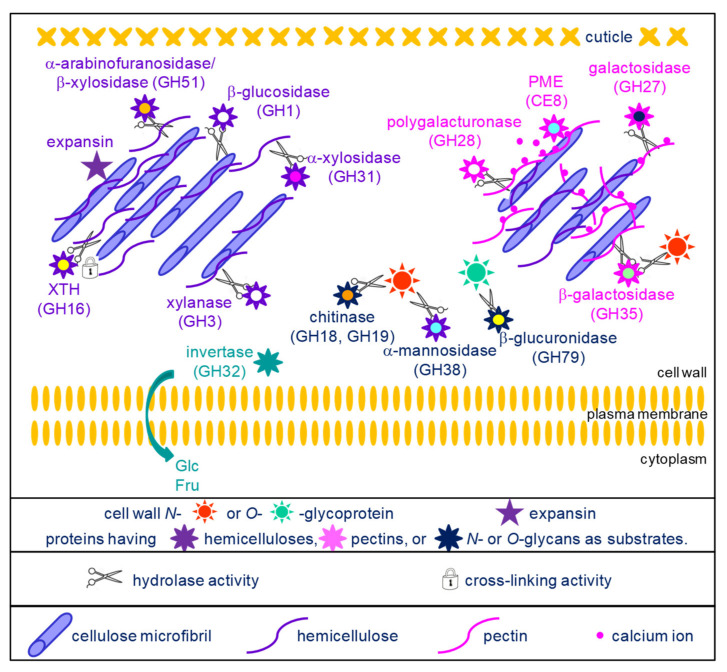
Schematic representation of the activities of the proteins acting on cell wall carbohydrates and belonging to the core cell wall proteome. With the exception of expansins and CE8, all the protein families are glycoside hydrolases (GHs) which were grouped according to their possible substrates (Section 4.1 for details): cellulose and hemicelluloses for GH1, GH3, GH16, GH31, GH51 and expansins (top left part of the scheme); pectins for GH27, GH28, GH35 and CE8 (top right part of the scheme); *N*- or *O*-glycans for GH18, GH19, GH38 and GH79 (center of the scheme); sucrose for GH32, thus releasing glucose (Glc) and fructose (Fru) which can be transferred to the cytoplasm by hexose transporters.

**Figure 3:**
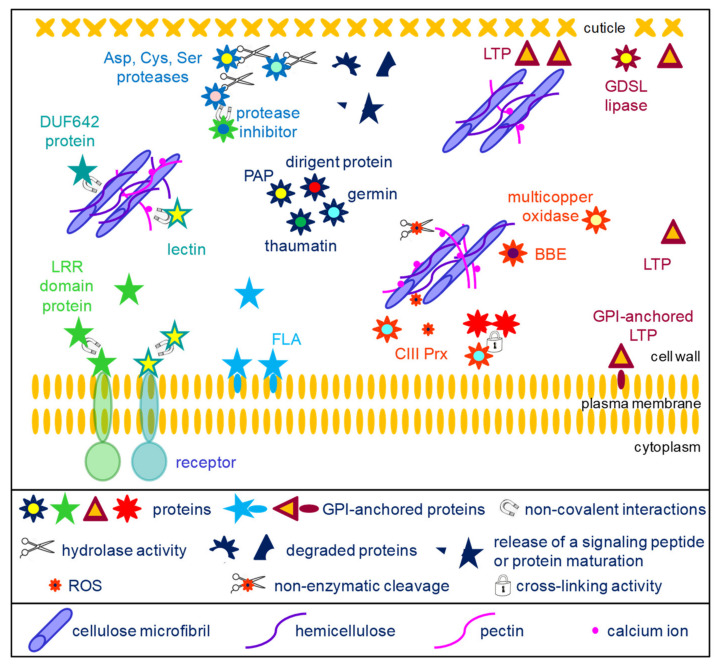
Schematic representation of the activities of diverse proteins belonging to the core cell wall proteome. The protein families have been grouped according to their known biological activities. Proteases are assumed to play roles in protein maturation, release of signaling peptides and protein degradation (top left of the scheme). DUF642 proteins and lectins interact with cell wall polysaccharides but their precise roles are not known (middle left part of the scheme). Several protein families could play roles in signaling (bottom left of the scheme): LRR proteins and lectins could interact with other proteins, and in particular with the extracellular domains of plasma membrane receptors, thus leading to the transduction of a signal to the cell; fasciclin arabinogalactan proteins (FLAs) are also assumed to play a role in signaling. Dirigent proteins, germins, thaumatins and purple acid phosphatases (PAPs) have diverse activities (center of the scheme, Section 4.2 for details). Oxido-reductases (multicopper oxidases, berberine-bridge oxido-reductases (BBEs) and class III peroxidases (CIII Prxs)) play multiple roles in the cell wall. In particular, CIII Prxs can cross-link structural proteins or phenolics compounds, and they contribute to the regulation of reactive oxygen species (ROS) which are involved in signaling or in non-enzymatic cleavage of polysaccharides (central part of the scheme). LTPs and GDSL lipases could play roles in the formation of cuticle (right side of the scheme). Some LTPs are localized at the surface of the plasma membrane thanks to GPI anchors and participate in the transport of lipids to the cuticle layer. LTPs have also been shown to play a role at the interface between the hydrophilic cell wall polysaccharides and the hydrophobic cuticle layer.

As mentioned, each of these functional classes includes several protein families. By comparing the 13 selected cell wall proteomes, it is possible to identify protein families which are present in all or in most of them (Appendix A). They are described in the two following paragraphs: proteins acting on cell wall carbohydrates belonging to the major functional class (Section 4.1, Figure 2) and proteins belonging to the other functional classes (Section 4.2, Figure 3).

### 4.1. Proteins Acting on Cell Wall Carbohydrates

These protein families can be distinguished on the basis of their carbohydrate substrates. They have been grouped according to their known or predicted substrates: hemicelluloses, pectins or glycans of glycoproteins (Figure 2).

A set of enzymes can act on hemicelluloses. GH16 are xyloglucan endotransglucosylases/hydrolases (XTHs). They were initially described as having xyloglucan-xyloglucan donor/acceptor substrate activities. However, it was later shown that they could accept other substrates such as cellulose or mixed-linkage (1,3;1,4)-β-D-glucans [95,96,97]. Molecular modelling had suggested that they could also modify arabinoxylans in *Poaceae* [97]. These findings allow assuming that they could play critical roles in remodeling the cellulose/hemicellulose networks in cell walls of both monocot and dicot plants. As an example, the *xth21* mutant of *A. thaliana* exhibited a dwarf phenotype most probably resulting from a defect in the growth of the primary root [98]. This mutant also showed a decrease in the average mass of xyloglucans and in cellulose content, suggesting the role of the cellulose/xyloglucan network in the elongation of the cell wall.

GH1 (mostly *β*-glucosidases), GH3 (xylanases), GH51 (arabinofuranosidases/*β*-xylosidases), GH31 (*α*-xylosidases) and GH17 (*β*-1,3-glucosidases) have a hydrolytic activity towards different types of hemicelluloses or callose [99]. *A. thaliana* mutants impaired in *AtBG_ppap* (*β*-1,3-glucanase_putative plasmodesmata-associated protein), have an increased amount of callose at the level of the plasmodesmata and the cell-to-cell movement of a fluorescent marker protein is slower than in wild type [100]. These results, together with identification of AtBG_ppap in a plasmodesmata proteome, suggest its role in the regulation of symplasmic communication.

Another group of enzymes can hydrolyze or modify pectin molecules. GH27 and GH28 hydrolyze galactomannans and homogalacturonans, respectively [99]. The *A. thaliana QRT3* (*QUARTET3*) gene was shown to encode a polygalacturonase and the corresponding mutant exhibited defect in pollen mother cell wall degradation resulting in the defect in microspore separation [101]. GH35 could act on the arabinan side-chains of pectins or on the *O*-glycans of AGPs although some of them could also act on xyloglucans [99]. PMEs operate the demethylesterification of homogalacturonans, thus revealing negative charges which allow the formation of the egg box structures with calcium ions [4]. The *A. thaliana atpme3* mutant was shown to have an increased number of adventitious roots together with an increase in the degree of HG methylesterification, thus suggesting the importance of changes in the pectin structure for adventitious root emergence [102].

Finally, a set of enzymes can hydrolyze the *N*- or the O-glycans of glycoproteins. They belong to GH families 18, 19 and 38 [103]. The *O*-glycans of AGPs were assumed to be substrates of GH19 as one of the few cell wall molecules carrying glucosamine or N-acetylglucosamine [104]. In the same article, it was shown that an incubation of an AGP fraction purified from carrot cells with an endochitinase of the GH19 family lead to the release of oligosaccharides. GH18 and GH19 were also described as chitinases/lysozymes playing roles during plant-microorganism interactions [105,106].

GH32 are cell wall acidic invertases. They cleave sucrose into glucose and fructose which can be uploaded by cells by hexose transporters. They are involved not only in phloem unloading and in the development of non-photosynthetic organs, but also in plant defense reactions [107,108].

### 4.2. The Other Conserved Protein Families

Apart from the proteins acting on cell wall carbohydrates, several protein families are also conserved (Figure 3). Several families of extracellular proteases are well conserved in cell wall proteomes, such as Asp proteases, Cys proteases and Ser proteases. The roles of these proteins have begun to be discovered in *A. thaliana*. The AtSBT1.4, AtSBT1.7 and AtSBT4.13 subtilisins were shown to release the signaling peptide CLE40 (Clavata3/Endosperm Surrounding Region 40) from a preprotein [109]. CLE40 is involved in the regulation of stem cell differentiation. Such extracellular proteases may also play roles in protein maturation as AtSBT1.6 for PMEs [83]. The SDD1 (Stomatal Density and Distribution 1) subtilisin negatively regulates the formation of stomata in *A. thaliana*, most probably through peptide signaling, although its substrate has not yet been identified [110]. Besides, the *A. thaliana* extracellular CDR1 (Constitutive Disease Resistance) Asp protease was assumed to mediate disease resistance through a signaling peptide [111]. Most probably, all these proteolytic activities are modulated by proteases inhibitors which are also found as conserved protein families in cell walls.

Among the ORs, CIII Prxs represent large plant gene families, with, for example, 73 members in *A. thaliana* and 189 in *M. polymorpha* (https://peroxibase.toulouse.inrae.fr) (accessed on 6 April 2022). They play major roles in plant cell walls by (i) generating reactive oxygen species (ROS) involved in signaling and in nonenzymatic cleavage of polysaccharides, or by regulating the level of H_2_O_2_, thus contributing to cell wall stiffening by cross-linking structural proteins such as extensins or monomers of lignins [9]. This latter role could also be played by laccases, such as LACCASE5 in *B. distachyon* culms [112]. Besides, an *A. thaliana* laccase (TRANSPARENT TESTA10) was shown to be involved in the polymerization of flavonoids in the seed coat [113]. The role of multicopper oxidases is more puzzling. The *A. thaliana SKU5* (SKEWED5) gene was shown to be involved in root directional growth [114]. Mutants impaired in *SKS11* and *SKS12* (*SKU SIMILAR11* and *12*) showed alteration in pollen tube integrity, growth and guidance as well as some alteration in polysaccharide composition [115]. No enzymatic activity has been demonstrated yet for the encoded proteins. Finally, the role of berberine-bridge enzyme-like proteins start to be understood thanks to the characterization of the enzymatic activity of the *A. thaliana* OGOX1-4 (oligogalacturonide OXIDASE 1-4) proteins [116]. They oxidize OGs which are less hydrolysable by fungal PGs and have reduced ability to activate immune response. However, no specific role has yet been demonstrated during plant development.

Several protein families related to lipid metabolism could be identified in most cell wall proteomes. Several roles have been proposed for non-specific lipid transfer proteins (LTPs) [117]. They have been assumed to contribute to the transfer of lipids which are hydrophobic molecules through the hydrophilic cell wall [118]. Indeed, *A. thaliana* mutants impaired in *LTPG2* or in *LTPG1* and *LTPG2* exhibit an alteration in cuticular wax composition in stems and siliques [119]. LTPG1 and LTPG2 are predicted to be GPI-anchored proteins. LTPs have also been shown to be involved in the adhesion of the cuticular layer on the hydrophilic primary cell wall [120]. Several roles were proposed for GDSL lipases/acylhydrolases [121]. The tomato GDSL1 was shown to be involved in the deposition of cutin in the cuticle of tomato fruits [122]. Indeed, the silencing of *GDSL1* leads to the appearance of nanopores in isolated fruit cutins and to a reduction in ester bond cross-links. An *A. thaliana* mutant impaired in *GELP77* exhibits shrunken pollen grains which stick together, suggesting a role of GELP77 in pollen grain wall formation [123]. More recently, GDSL lipases/acylhydrolases were assumed to also be involved in suberin degradation [124].

Among the miscellaneous proteins, dirigent proteins (DIRs) are assumed to be involved in lignan and in lignin biosynthesis. They have no known enzymatic activity, but they would control the regio- and stereoselectivity of bimolecular phenoxy radical coupling [91]. As an example, the *A. thaliana* AtDIR10 protein was shown to be essential for the establishment of the lignin-based Casparian strips in roots [125]. Several types of enzymatic activities have been associated to germins and germin-like proteins: manganese superoxide dismutase (SOD), oxalate oxidase (OXO) or ADP glucose pyrophosphatase/phosphodiesterase (AGPPase) [126,127]. Thaumatins and thaumatin-like proteins belong to the large pathogenesis-related protein family (PR proteins) and are also called PR-5 [128]. Most of them exhibit an anti-fungal activity and their genes are induced upon biotic stress. They might also have allergenic properties. Extracellular purple acid phosphatases (PAPs) are phosphohydrolases able to cleave Pi from organic Pi-esters that are inaccessible to root cells in soils, for example [92]. The predominant *A. thaliana* PAPs (AtPAP12 and AtPAP26) were identified in several cell wall proteomes [22,31,32,129] and both proteins were isolated from the culture medium of cell suspensions cultures [130].

Fasciclin arabinogalactan proteins (FLAs) are assumed to be involved in the interactions between the cells and their environment in the same way as mammalian proteins carrying fasciclin domains (FAS1) [131]. Some of them are located at the plasma membrane surface thanks to the presence of a GPI-anchor as experimentally demonstrated for AtFLA4 and AtFLA12 [132,133]. They could also be released in the cell wall after GPI-anchor cleavage. AtFLA4 was assumed to interact with pectin molecules and to contribute to the biomechanical properties of the cell wall [131]. FLAs were also found to be present in the so-called G-layer of tension wood. In particular, mutants impaired in AtFLA11 and AtFLA12 exhibit reduced tensile strength and stiffness [134]. In this case, interactions between FLAs and cellulose microfibrils were suspected. Furthermore, in the functional class comprising signaling molecules, proteins with leucine-rich repeats (LRRs) are found in all cell wall proteomes. Their role is not clear but they could interact with other proteins or with peptides. Such interactions have been reported for the LRR domains of AtLRX2 and AtLRX8 interacting with the rapid alkalinization factor 4 (RALF4) signaling peptide [135].

The DUF 642 (domain of unknown function 642, InterPro domain IPR006946) proteins were initially identified as major proteins in the cell wall proteome of *A. thaliana* etiolated hypocotyls [31]. The DUF 642 domain is frequently associated with a galactose-binding-like domain (InterPro domain IPR008979). Different roles were proposed, such as a structural role as lectin-like proteins interacting with cell wall polysaccharides [136] or a role in the regulation of PME activity [137].

## 5. What about the Non-Canonical Proteins Identified in Cell Wall Proteomes?

All the published proteomes characterized from purified cell walls, extracellular fluids or cell suspension culture media contain proteins which are not expected to be secreted. These proteins have now been included in a new version of the plant cell wall proteome database called *WallProtDB-2* (https://www.polebio.lrsv.ups-tlse.fr/WallProtDB/) (accessed on 6 April 2022) to allow obtaining an overview of their predicted sub-cellular localization and biological activity. Apart from the 4292 proteins considered to be bona fide CWPs (Section 3), *WallProtDB-2* now contains 6462 proteins presumed to be intracellular and identified in apoplastic fluids or among proteins extracted from purified cell walls (Table 2). These proteins are assumed to be non-canonical CWPs. To our knowledge, this is the first time that this information has been collected.

In the following, 12 cell wall proteomes have been taken into account (Table 2). Altogether, they comprise 6425 presumed contaminants proteins. The *B. oleracea* and the *P. patens* proteomes have been excluded because the former is a xylem sap proteome and the latter is very small one.

**Figure 4:**
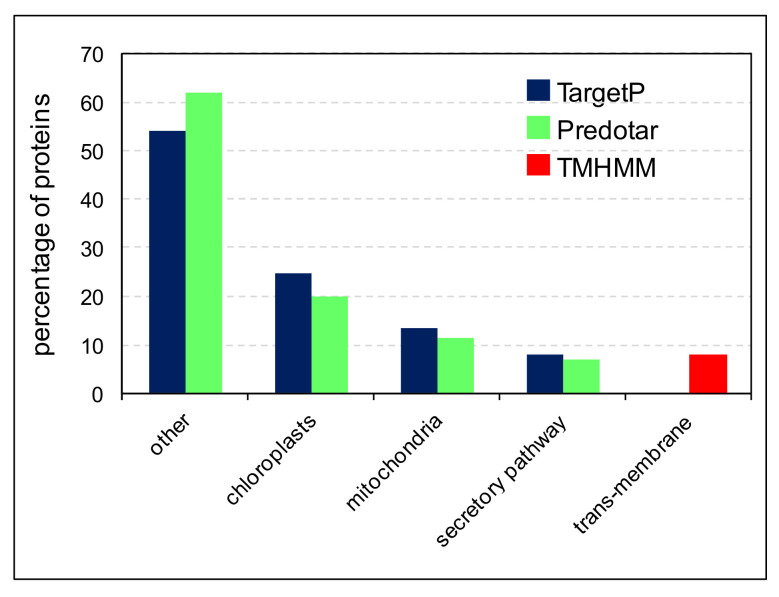
Prediction of the sub-cellular localization of the proteins presumed to be contaminants in cell wall proteomes. The predictions were made with TargetP, Predotar or TMHMM. “Trans-membrane” stands for trans-membrane domains.

Using TargetP (https://services.healthtech.dtu.dk/service.php?TargetP-2.0) (accessed on 6 April 2022) and Predotar (https://urgi.versailles.inrae.fr/predotar/) (accessed on 6 April 2022), the proteins presumed to be contaminants were predicted to be targeted to chloroplasts (between 19.9 and 24.8%), mitochondria (between 11.3 and 13.3%), the secretory pathway (between 6.9 and 7.8%) and other cell compartments (between 54.0 and 61.7%) (Figure 4). In the case of proteins predicted to be targeted to the secretory pathway, some of them have ER retention signals, or multiple transmembrane-domains such as transporters (7.8% as predicted by TMHMM, https://services.healthtech.dtu.dk/service.php?TMHMM-2.0) (accessed on 6 April 2022).

**Figure 5:**
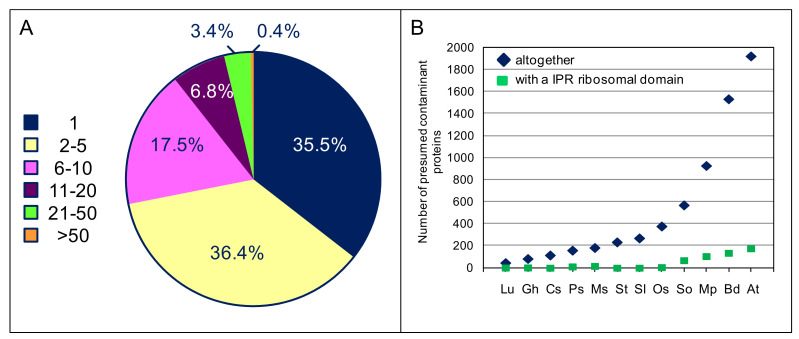
Overview of the distribution of Pfam domains in the presumed contaminant proteins of the 12 studied cell wall proteomes. (**A**) This pie chart represents the percentage of IPR domains shared by a given number of presumed contaminant proteins in the overall population of 6423 presumed contaminant proteins (from 1 to more than 50). Note that a given protein can contain several domains. For example, 559 Pfam domains (35.5%) are only present in one protein, whereas 6 domains (0.4%) are shared by more than 50 proteins. (**B**) This graph represents the total number of presumed contaminant proteins in each of the cell wall proteomes present in *WallProtDB-2* (dark blue diamonds) and the number of proteins exhibiting a ribosomal domain (green squares). See Appendix A. for details. Lu: *L. usitatissimum*; Gh: *G. hirsutum*; Cs: *C. sinensis*; Ps: *Populus* spp.; Ms: *M. sativa*; St: *S. tuberosum*; Sl: *S. lycopersicum*; Os: *O. sativa*; So: *S. officinarum*; Mp: *M. polymorpha*; Bd: *B. distachyon*; At: *A. thaliana*.

A very high number of domains could be predicted in the proteins presumed to be contaminant: 1575 Pfam (https://xfam.org/) (accessed on 6 April 2022) and 3024 IPR (https://www.ebi.ac.uk/interpro/) (accessed on 6 April 2022) domains (Appendix A). This result shows the huge diversity of these proteins. One third of the Pfam domains (560) were only present in one protein whereas 6 domains were shared by more than 50 proteins (Figure 5A). Similar results were observed for IPR domains with 938 domains (about one third) only present in one protein and 36 domains present in more than 50 proteins (Appendix A). The number of proteins sharing a given domain increases with the number of presumed contaminants in a given cell wall proteome. Figure 5B illustrates the case of proteins predicted to have a IPR ribosomal domain. Among these domains, there are (i) structural domains such as PF00076 (RNA recognition motif) shared by 174 proteins and IPR016040 (NAD(P)-binding domain) shared by 315 proteins or (ii) domains corresponding to a biological activity such as PF00012 (Hsp70 family) shared by 67 proteins, and IPR013766 (thioredoxin domain) shared by 159 proteins (Appendix A). The top 20 most represented Pfam domains describing a biological activity are listed in Table 3. None of these functions have already been described in the extracellular space.

The frequent identification of certain proteins in cell wall proteomes may have different explanations: (i) they could exhibit specific features allowing them to strongly interact with cell wall components during the purification of cell walls, for example, the histones (61 entries in 7 plant species, PF00125, IPR007125), which are basic proteins like most CWPs [12]; (ii) they could be very abundant proteins such as ribosomal proteins (altogether 578 entries in 8 plant species); or (iii) secreted through alternative secretory pathways. For some protein families, there is no clear hypothesis regarding their presence in many cell wall proteomes: e.g., thioredoxin (e.g., PF00085 with 117 occurrences in 12 plant species), heat-shock proteins (e.g., PF00012 with 67 proteins in 12 plant species), glyceraldehyde 3-phosphate dehydrogenase (PF02800 and PF00044 with 46 and 45 proteins in 11 and 10 plant species, respectively), lactate/malate dehydrogenase (PF02866 and PF00056 with 49 proteins in 10 plant species) and cyclophilin type peptidyl-prolyl cis-trans isomerase (42 proteins in 9 plant species). Finally, these proteins could be moonlighting ones, being present in different cell compartments and having different functions in each of them [138]. As an example, two non-specific lipid transfer proteins of *A. thaliana*, AtLTP2 and AtLTP4, have been localized in both the cell wall and chloroplasts [120,132].

As mentioned above, UPS pathways have been described in bacteria and mammals. In plants, the best documented example of the presence of leaderless proteins in the apoplast is probably that of the leaderless jacalin-related lectin of *Helianthus annuus* (Helja): it has been identified in extracellular fluids [139], and in extracellular vesicles [140], and it has been immunolocalized in the extracellular matrix [139]. Another example is that of the cytoplasmic mannitol dehydrogenase which has been immunolocalized in cell walls upon a salicylic treatment [23].

As for mammalian cells, four main UPS pathways have been proposed in plants [13]: a direct ER to plasma membrane traffic, plasma membrane transporter channels, secretory lysosomes, and multivesicular bodies (MVBs) leading to exosome secretion. Besides, exocyst positive organelles (EXPOs) with a double membrane have been characterized in *A. thaliana* and in *Nicotiana tabacum* cells [141]. Exocysts are proteins mediating the fusion between post-Golgi vesicles and the plasma membrane, thus allowing the release of proteins in the extracellular space. All these pathways are resistant to brefeldin A which disrupts the ER-Golgi vesicular traffic. However, it must be stressed that additional work has to be done to better define what is presently called extracellular vesicles (EVs) and to identify specific markers to allow comparing different studies [142].

Recent research has been devoted to EVs in *A. thaliana* and *H. annuus* upon pathogen infection or in response to salicylic acid treatment [140,143], and in *Nicotiana benthamiana* upon viral infection [144]. These vesicles contain proteins involved in plant defense reactions, in membrane trafficking; among which are proteins with or without predicted signal peptides. They have also been shown to deliver small RNAs to fungal pathogens [145] and viral components in the cell wall [144]. Whether these EVs are EXPOs and whether plants produce different kinds of EVs remain to be determined [142,146].

Unfortunately, no bioinformatic program similar to SecretomeP has yet been designed for plant proteins (Section 3). In this bioinformatic program, it is assumed that proteins present in extracellular spaces share common features whatever the route of secretion [14]. Such a tool would be useful to help sort the proteins devoid of a predictable signal peptide and focusing experimental work on them to demonstrate their actual presence in apoplastic fluids or in cell walls.

## 6. Conclusions

Altogether, the large amount of data accumulated during the last twenty years allows drawing a detailed picture of the cell wall proteome. A set of conserved protein families is present in all of them. Besides, the composition of the cell wall depends on the plant species, with differences between bryophytes, *Poaceae* and dicots [1,6]. However, the same protein families can be identified in all the cell wall proteomes characterized thus far. The current hypothesis is that they are either required for basic cell wall functions, quick answers to environmental stresses or in combination. As shown in this article, this collection of CWPs could (i) manage the rearrangement of the networks of cell wall polysaccharides; (ii) contribute to protein turnover, protein maturation of release of biologically active peptides; or (iii) play roles in signaling. In addition, they may be involved in the regulation of the symplastic transport. Studying additional cell wall proteomes would contribute to obtaining an even more precise description of the core proteome and scale it down to the organ level.

The question of the presence of unexpected leaderless proteins, the non-canonical proteins, in cell wall proteomes need to be further examined with a more precise description of the extracellular vesicles mostly observed upon pathogen infections. Additional experimental work has to be performed to demonstrate the presence of the unexpected proteins in extracellular spaces with their detection with specific antibodies or sub-cellular localization using fluorescent proteins. It is doubtful that all these proteins are *bona fide* CWPs. Many of them are most probably present as contaminants since the procedures used to extract extracellular fluids or to purify cell walls exhibit many drawbacks, notably due to the fact that the cell wall is an open compartment. The information provided in this article regarding the proteins families identified in most cell wall proteomes can provide clues to select candidates for testing their actual sub-cellular localization.

The next challenges for the cell wall proteomics studies will be a better description of the CWP post-translational modifications, a better knowledge of protein half-lives, and the design of methods to increase the cell wall coverage. Indeed, the known cell wall proteomes lack heavily *O*-glycosylated proteins, such as AGPs, or covalently-linked proteins, such as extensins or proline-rich proteins. Besides, peptidomics have to be developed to obtain an extensive description of the peptides present in cell walls which are key to understanding the signaling mechanisms through cell walls which are involved in developmental processes and responses to environmental cues [89]. Finally, the integration of transcriptomics and proteomics data will be critical to fully understanding the fine regulation of expression of the genes encoding CWPs.

## Figures and Tables

**Table 1:** The plant cell wall proteomes analyzed in this study.

Plant Species (Number of Identified CWPs)	Organ	Method ^a^	References
*Marchantia polymorpha* (409)	thallus	cell wall isolation (3)	[29]
	thallus	*N*-glycoproteome (4)	[29]
*Arabidopsis thaliana* (992)	etiolated hypocotyl root	cell wall isolation (3) cell wall isolation (3)	[30,31] [32]
	rosette rosette stem stem cell suspension culture cell suspension culture etiolated seedling	vacuum infiltration (2) cell wall isolation (3) cell wall isolation (3) *N*-glycoproteome (4) cell wall isolation (3) culture medium (1) culture medium (1)	[33] [21,22,34] [22,35] [36] [37,38] [23,25,26] [39]
*Linum usitatissimum* (106)	stem	cell wall isolation (3)	[40]
*Medicago sativa* (322)	stem	cell wall isolation (3)	[41,42]
*Populus spp.* (143)	leaf stem	vacuum infiltration (2) vacuum infiltration (2)	[43] [43]
*Solanum lycopersicum* (187)	fruit pericarp fruit cuticle	*N*-glycoproteome (4) vacuum infiltration (2)	[44] [45]
*Solanum tuberosum* (205)	leaf	cell wall isolation (3)	[24,46]
*Gossypium hirsutum* (139)	seed	cell wall isolation (3)	[47]
*Camellia sinensis* (267)	leaf leaf	cell wall isolation (3) *N*-glycoproteome (4)	[48] [48]
*Saccharum officinarum* (275)	leaf leaf stem stem cell suspension culture	vacuum infiltration (2) cell wall isolation (3) vacuum infiltration (2) cell wall isolation (3) cell wall isolation (3)	[49,50] [50] [49,51] [51] [52]
*Triticum aestivum* (636)	seed	cell wall isolation (3)	[27]
*Oryza sativa* (322)	root	cell wall isolation (3)	[53]
	leaf	cell wall isolation (3)	[54]
	cell suspension culture	cell wall isolation (3)	[55]
	callus	culture medium (1)	[54,55]
*Brachypodium distachyon* (721)	seed seedling	cell wall isolation (3) *N*-glycoproteome (4)	[56,57] [58]
	leaf stem	cell wall isolation (3) cell wall isolation (3)	[20,59,60] [59,60]

^a.^ Numbers 1–4 refer to the protocol used to study the extracellular proteome (Figure 1).

**Table 2:** An overview of the proteins present in *WallProtDB-2*.

Plant Species	CWPs (Percentage of CWPs among All the Identified Proteins)	Proteins Presumed to Be Contaminant Proteins
*Marchantia polymorpha*	409 (30.6%)	928
*Physcomitrella patens* ^a^	19 (57.6%)	14
*Arabidopsis thaliana*	992 (34.1%)	1924
*Brassica oleracea* ^b^	162 (85.7%)	27
*Linum usitatissimum*	106 (69.7%)	46
*Medicago sativa*	322 (63.8%)	183
*Populus* spp.	143 (47.4%)	159
*Solanum lycopersicum*	187 (40.7%)	268
*Solanum tuberosum*	205 (46.7%)	234
*Gossypium hirsutum*	139 (62.6%)	83
*Camellia sinensis*	267 (69.9%)	115
*Saccharum officinarum*	275 (32.5%)	570
*Oryza sativa*	345 (47.7%)	378
*Brachypodium distachyon*	721 (32.0%)	1534

^a.^ The proteome of *P. patens* has not been included in this study because of its small size. ^b.^ The proteome of *B. oleracea* has not been considered in this work since this is a xylem sap proteome.

**Table 3:** The most represented Pfam domains in the proteins presumed to be contaminants in at least 9 out of the 12 studied cell wall proteomes.

Pfam Domain	Domain Name	Number of Occurrences	Number of Plant Species
PF00085	thioredoxin	117	12
PF13848	thioredoxin-like domain	33	11
PF00012	Hsp70 protein	67	12
PF02800	glyceraldehyde 3-phosphate dehydrogenase, C-terminal domain	46	11
PF00044	glyceraldehyde 3-phosphate dehydrogenase, NAD binding domain	45	10
PF02866	lactate/malate dehydrogenase, alpha/beta C-terminal domain	49	10
PF00056	lactate/malate dehydrogenase, NAD binding domain	49	10
PF00274	fructose-bisphosphate aldolase class-I	32	10
PF00903	glyoxalase/bleomycin resistance protein/dioxygenase superfamily	26	10
PF00121	triosephosphate isomerase	23	10
PF03952	enolase, N-terminal domain	23	10
PF00113	enolase, C-terminal TIM barrel domain	23	10
PF08267	cobalamin-independent synthase, N-terminal domain	19	10
PF01717	cobalamin-independent synthase, catalytic domain	18	10
PF00227	proteasome subunit	47	9
PF00160	cyclophilin type peptidyl-prolyl cis-trans isomerase/CLD	42	9
PF07992	pyridine nucleotide-disulphide oxidoreductase	42	9
PF00155	aminotransferase class I and II	31	9
PF00262	calreticulin family	23	9
PF13417	glutathione S-transferase, N-terminal domain	18	9
PF00658	poly-adenylate binding protein, unique domain	15	9
PF00347	ribosomal protein L6	14	9
PF05757	oxygen evolving enhancer protein 3 (PsbQ)	13	9
PF02789	cytosol aminopeptidase family, N-terminal domain	11	9
PF00883	cytosol aminopeptidase family, catalytic domain	11	9
PF00227	Proteasome subunit	47	9

## Data Availability

With the exception of the *T. aestivum* data ([23]), the wall proteomics data can be found at *WallProtDB-2* (http://www.polebio.lrsv.ups-tlse.fr/WallProtDB/) (accessed on 6 April 2022) with the references to the corresponding articles.

## Supplementary Materials

The following supporting information can be downloaded at: https://www.mdpi.com/article/10.3390/ijms23084273/s1.

## Appendix A

The core cell wall proteome (Table S1).

Prediction of the sub-cellular localization of the presumed contaminant proteins (IPR and Pfam domains) (Table S2).

## References

[1] Carpita N.C., Gibeaut D.M. (1993). Structural models of primary cell walls in flowering plants, consistency of molecular structure with the physical properties of the walls during growth. Plant J..

[2] Mnich E., Bjarnholt N., Eudes A., Harholt J., Holland C., Jørgensen B., Larsen F., Liu M., Manat R., Meyer A. (2020). Phenolic cross-links: Building and de-constructing the plant cell wall. Nat. Prod. Rep..

[3] Caffall K., Mohnen D. (2009). The structure, function, and biosynthesis of plant cell wall pectic polysaccharides. Carbohydr. Res..

[4] Hocq L., Pelloux J., Lefebvre V. (2017). Connecting homogalacturonan-type pectin remodeling to acid growth. Trends Plant Sci..

[5] Scheller H.V., Ulvskov P. (2010). Hemicelluloses. Ann. Rev. Plant Biol..

[6] Popper Z., Fry J. (2003). Primary cell wall composition of Bryophytes and Charophytes. Ann. Bot..

[7] Carpita N. (2011). Update on mechanisms of plant cell Wall biosynthesis: How plants make cellulose and other (1→4)-β-D-glycans. Plant Physiol..

[8] Park A., Cosgrove D. (2015). Xyloglucan and its Interactions with other components of the growing cell wall. Plant Cell. Physiol..

[9] Francoz E., Ranocha P., Nguyen-Kim H., Jamet E., Burlat V., Dunand C. (2015). Roles of cell wall peroxidases in plant development. Phytochemistry.

[10] Schardon K., Hohl M., Graff L., Pfannstiel J., Schulze W., Stintzi A., Schaller A. (2016). Precursor processing for plant peptide hormone maturation by subtilisin-like serine proteinases. Science.

[11] Yang Y., Anderson C., Cao J. (2021). Polygalacturonase45 cleaves pectin and links cell proliferation and morphogenesis to leaf curvature in *Arabidopsis thaliana*. Plant J..

[12] Jamet E., Albenne C., Boudart G., Irshad M., Canut H., Pont-Lezica R. (2008). Recent advances in plant cell wall proteomics. Proteomics.

[13] Rose J.K.C., Lee S.-J. (2010). Straying off the highway: Trafficking of secreted plant proteins and complexity in the plant cell wall proteome. Plant Physiol..

[14] Bendtsen J.D., Jensen L.J., Blom N., von Heijne G., Brunak S. (2004). Feature based prediction of non-classical and leaderless protein secretion. Protein Eng. Des. Sel..

[15] Bendtsen J., Kiemer L., Fausbøll A., Brunak S. (2005). Non-classical protein secretion in bacteria. BMC Microbiol..

[16] Albenne C., Canut H., Jamet E. (2013). Plant cell wall proteomics: The leadership of *Arabidopsis thaliana*. Front. Plant Sci..

[17] Initiative A.G. (2000). Analysis of the genome sequence of the flowering plant Arabidopsis thaliana. Nature.

[18] Aebersold R., Mann M. (2016). Mass-spectrometric exploration of proteome structure and function. Nature.

[19] Albenne C., Canut H., Hoffmann L., Jamet E. (2014). Plant cell wall proteins: A large body of data, but what about runaways?. Proteomes.

[20] Pinski A., Betekhtin A., Skupien-Rabian B., Jankowska U., Jamet E., Hasterok R. (2021). Changes in the cell wall proteome of leaves in response to high etmperature stress in *Brachypodium distachyon*. Int. J. Mol. Sci..

[21] Duruflé H., Hervé V., Ranocha P., Balliau T., Zivy M., Chourré J., San Clemente H., Burlat V., Albenne C., Déjean S. (2017). Cell wall adaptation of two contrasted ecotypes of *Arabidopsis thaliana*, Col and Sha, to sub-optimal growth conditions: An integrative study. Plant Sci..

[22] Duruflé H., Ranocha P., Balliau T., Dunand C., Jamet E. (2019). Transcriptomic and cell wall proteomic datasets of rosettes and floral stems from five *Arabidopsis thaliana* ecotypes grown at optimal or sub-optimal temperature. Data Brief.

[23] Cheng F., Zamski E., Guo W., Pharr D., Williamson J. (2009). Salicylic acid stimulates secretion of the normally symplastic enzyme mannitol dehydrogenase: A possible defense against mannitol-secreting fungal pathogens. Planta.

[24] Bengtsson T., Weighill D., Levander F., Resjö S., Burra D., Moushib L., Hedley P., Liljeroth E., Jacobson D., Alexandersson E. (2014). Proteomics and transcriptomics of the BABA-induced resistance response in potato using a novel functional annotation approach. BMC Genom..

[25] Tran H., Plaxton W. (2008). Proteomic analysis of alterations in the secretome of *Arabidopsis thaliana* suspension cells subjected to nutritional phosphate deficiency. Proteomics.

[26] Oh I.S., Park A.R., Bae M.S., Kwon S.J., Kim Y.S., Lee J.E., Kang N.Y., Lee S., Cheong H., Park O.K. (2005). Secretome analysis reveals an Arabidopsis lipase involved in defense against Alternaria brassicicola. Plant Cell.

[27] Cherkaoui M., Lollier V., Geairon A., Bouder A., Larré C., Rogniaux H., Jamet E., Guillon F., Francin-Allami M. (2020). Cell wall proteome of wheat grain endosperm and outer layers at two key stages of early development. Int. J. Mol. Sci..

[28] San Clemente H., Jamet E. (2015). *WallProtDB*, a database resource for plant cell wall proteomics. Plant Methods.

[29] Kolkas H., Balliau T., Chourré J., Zivy M., Canut H., Jamet E. (2022). The cell wall proteome of *Marchantia polymorpha* reveals specificities compared to those of flowering plants. Front. Plant Sci..

[30] Feiz L., Irshad M., Pont-Lezica R.F., Canut H., Jamet E. (2006). Evaluation of cell wall preparations for proteomics: A new procedure for purifying cell walls from Arabidopsis hypocotyls. Plant Methods.

[31] Irshad M., Canut H., Borderies G., Pont-Lezica R., Jamet E. (2008). A new picture of cell wall protein dynamics in elongating cells of *Arabidopsis thaliana*: Confirmed actors and newcomers. BMC Plant Biol..

[32] Nguyen-Kim H., San Clemente H., Balliau T., Zivy M., Dunand C., Albenne C., Jamet E. (2016). *Arabidopsis thaliana* root cell wall proteomics: Increasing the proteome coverage using a combinatorial peptide ligand library and description of unexpected Hyp in peroxidase amino acid sequences. Proteomics.

[33] Boudart G., Jamet E., Rossignol M., Lafitte C., Borderies G., Jauneau A., Esquerré-Tugayé M.-T., Pont-Lezica R. (2005). Cell wall proteins in apoplastic fluids of *Arabidopsis thaliana* rosettes: Identification by mass spectrometry and bioinformatics. Proteomics.

[34] Hervé V., Duruflé H., San Clemente H., Albenne C., Balliau T., Zivy M., Dunand C., Jamet E. (2016). An enlarged cell wall proteome of *Arabidopsis thaliana* rosettes. Proteomics.

[35] Duruflé H., San Clemente H., Balliau T., Zivy M., Dunand C., Jamet E. (2017). Cell wall proteome analysis of *Arabidopsis thaliana* mature stems. Proteomics.

[36] Minic Z., Jamet E., Negroni L., der Garabedian P.A., Zivy M., Jouanin L. (2007). A sub-proteome of *Arabidopsis thaliana* trapped on Concanavalin A is enriched in cell wall glycoside hydrolases. J. Exp. Bot..

[37] Borderies G., Jamet E., Lafitte C., Rossignol M., Jauneau A., Boudart G., Monsarrat B., Esquerré-Tugayé M.T., Boudet A., Pont-Lezica R. (2003). Proteomics of loosely bound cell wall proteins of *Arabidopsis thaliana* cell suspension cultures: A critical analysis. Electrophoresis.

[38] Chivasa S., Ndimba B.K., Simon W.J., Robertson D., Yu X.-L., Knox J.P., Bolwell P., Slabas A.R. (2002). Proteomic analysis of the *Arabidopsis thaliana* cell wall. Electrophoresis.

[39] Charmont S., Jamet E., Pont-Lezica R., Canut H. (2005). Proteomic analysis of secreted proteins from *Arabidopsis thaliana* seedlings: Improved recovery following removal of phenolic compounds. Phytochemistry.

[40] Day A., Fénart S., Neutelings G., Hawkins S., Rolando C., Tokarski C. (2013). Identification of cell wall proteins in the flax (*Linum usitatissimum*) stem. Proteomics.

[41] Verdonk J.C., Hatfield R.D., Sullivan M.L. (2012). Proteomic analysis of cell walls of two developmental stages of alfalfa stems. Front. Plant Sci..

[42] Printz B., Dos Santos Morais R., Wienkoop S., Sergeant K., Lutts S., Hausman J.F., Renaut J. (2015). An improved protocol to study the plant cell wall proteome. Front. Plant Sci..

[43] Pechanova O., Hsu C.Y., Adams J.P., Pechan T., Vandervelde L., Drnevich J., Jawdy S., Adeli A., Suttle J.C., Lawrence A.M. (2010). Apoplast proteome reveals that extracellular matrix contributes to multistress response in poplar. BMC Genom..

[44] Catalá C., Howe K., Hucko S., Rose J., Thannhauser T. (2011). Towards characterization of the glycoproteome of tomato (*Solanum lycopersicum*) fruit using Concanavalin A lectin affinity chromatography and LC-MALDI-MS/MS analysis. Proteomics.

[45] Yeats T., Howe K., Matas A., Buda G., Thannhauser T., Rose J. (2010). Mining the surface proteome of tomato (*Solanum lycopersicum*) fruit for proteins associated with cuticle biogenesis. J. Exp. Bot..

[46] Lim S., Chisholm K., Coffin R.H., Peters R.D., Al-Mughrabi K.I., Wang-Pruski G., Pinto D.M. (2012). Protein profiling in potato (*Solanum tuberosum* L.) leaf tissues by differential centrifugation. J. Proteome Res..

[47] Kumar S., Kumar K., Pandey P., Rajamani V., Padmalatha K., Dhandapani G., Kanakachari M., Leelavathi S., Kumar P., Reddy V. (2013). Glycoproteome of elongating cotton fiber cells. Mol. Cell. Proteom..

[48] Liu Y., Ma L., Cao D., Gong Z., Fan J., Hu H., Jin X. (2021). Investigation of cell wall proteins of *C. sinensis* leaves by combining cell wall proteomics and N-glycoproteomics. BMC Plant Biol..

[49] Fonseca J., Calderan-Rodrigues M.J., de Moraes F., Cataldi T., Jamet E., Labate C. (2018). Cell wall proteome of sugarcane young and mature leaves and stems. Proteomics.

[50] Calderan-Rodrigues M., Guimarães Fonseca J., De Moraes F., Vaz Setem L., Carmanhanis Begossi A., Labate C. (2019). Plant cell wall proteomics: A focus on monocot species, *Brachypodium distachyon*, *Saccharum* spp., and *Oryza sativa*. Int. J. Mol. Sci..

[51] Calderan-Rodrigues M.J., Jamet E., Douché T., Rodrigues Bonassi M.B., Regiani Cataldi T.R., Guimaraes Fonseca J.G., San Clemente H., Pont-Lezica R., Labate C.A. (2016). Cell wall proteome of sugarcane stems: Comparison of a destructive and a non-destructive extraction method showed differences in glycoside hydrolases and peroxidases. BMC Plant Biol..

[52] Calderan-Rodrigues M., Jamet E., Calderan Rodrigues Bonassi M., Guidetti-Gonzalez S., Carmanhanis Begossi A., Vaz Setem L., Franceschini L., Guimarães Fonseca J., Labate C. (2014). Cell wall proteomics of sugarcane cell suspension cultures. Proteomics.

[53] Zhou L., Bokhari S.A., Dong C.J., Liu J.Y. (2011). Comparative proteomics analysis of the root apoplasts of rice seedlings in response to hydrogen peroxide. PLoS ONE.

[54] Jung Y.H., Jeong S.H., Kim S.H., Singh R., Lee J.E., Cho Y.S., Agrawal G.K., Rakwal R., Jwa N.S. (2008). Systematic secretome analyses of rice leaf and seed callus suspension-cultured cells: Workflow development and establishment of high-density two-dimensional gel reference maps. J. Proteome Res..

[55] Cho W.K., Chen X.Y., Chu H., Rim Y., Kim S., Kim S.T., Kim S.W., Park Z.Y., Kim J.Y. (2009). The proteomic analysis of the secretome of rice calli. Physiol. Plant..

[56] Francin-Allami M., Merah K., Albenne C., Rogniaux H., Pavlovic M., Lollier V., Sibout R., Guillon F., Jamet E., Larré C. (2015). Cell wall proteomics of *Brachypodium distachyon* grains: A focus on cell wall remodeling proteins. Proteomics.

[57] Francin-Allami M., Lollier V., Pavlovic M., San Clemente H., Rogniaux H., Jamet E., Guillon F., Larré C. (2016). Understanding the remodeling of cell walls during *Brachypodium distachyon* grain development through a sub-cellular quantitative proteomic approach. Proteomes.

[58] Zhang M., Chen G., Lv D., Li X., Yan Y. (2015). *N*-linked glycoproteome profiling of seedling leaf in *Brachypodium distachyon* L.. J. Proteome Res..

[59] Douché T., San Clemente H., Burlat V., Roujol D., Valot B., Zivy M., Pont-Lezica R., Jamet E. (2013). *Brachypodium distachyon* as a model plant toward improved biofuel crops: Search for secreted proteins involved in biogenesis and disassembly of cell wall polymers. Proteomics.

[60] Douché T., Valot B., Balliau T., San Clemente H., Zivy M., Jamet E. (2021). Cell wall proteomic datasets of stems and leaves of *Brachypodium distachyon*. Data Brief.

[61] Faye L., Boulaflous A., Benchabane M., Gomord V., Michaud D. (2005). Protein modifications in the plant secretory pathway: Current status and practical implications in molecular pharming. Vaccine.

[62] Armenteros J., Salvatore M., Winther O., Emanuelsson O., von Heijne G., Elofsson A., Nielsen H. (2019). Detecting sequence signals in targeting peptides using deep learning. Life Sci. Alliance.

[63] Armenteros J., Tsirigos K., Sønderby C., Petersen T., Winther O., Brunak S., von Heijne G., Nielsen H. (2019). SignalP 5.0 improves signal peptide predictions using deep neural networks. Nat. Biotechnol..

[64] Käll L., Krogh A., Sonnhammer E. (2004). A combined transmembrane topology and signal peptide prediction method. J. Mol. Biol..

[65] Small I., Peeters N., Legeai F., Lurin C. (2004). Predotar: A tool for rapidly screening proteomes for N-terminal targeting sequences. Proteomics.

[66] Goldberg T., Hecht M., Hamp T., Karl T., Yachdav G., Ahmed N., Altermann U., Angerer P., Ansorge S., Balasz K. (2014). LocTree3 prediction of localization. Nucleic Acids Res..

[67] Schwacke R., Schneider A., Van der Graaff E., Fischer K., Catoni E., Desimone M., Frommer W., Flugge U., Kunze R. (2003). ARAMEMNON, a novel database for Arabidopsis integral membrane proteins. Plant Physiol..

[68] Hofmann K., Stoffel W. (1993). TMbase—A database of membrane spanning proteins segments. Biol. Chem. Hoppe-Seyler.

[69] Sonnhammer E.L., von Heijne G., Krogh A. (1998). A hidden Markov model for predicting transmembrane helices in protein sequences. Proc. Int. Conf. Intell. Syst. Mol. Biol..

[70] Pierleoni A., Martelli P.L., Casadio R. (2008). PredGPI: A GPI-anchor predictor. BMC Bioinform..

[71] Fankhauser N., Mäser P. (2005). Identification of GPI anchor attachment signals by a Kohonen self-organizing map. Bioinformatics.

[72] San Clemente H., Pont-Lezica R., Jamet E. (2009). Bioinformatics as a tool for assessing the quality of sub-cellular proteomic strategies and inferring functions of proteins: Plant cell wall proteomics as a test case. Bioinform. Biol. Insights.

[73] Bellande K., Bono J.-J., Savelli B., Jamet E., Canut H. (2017). Plant lectins and lectin receptor-like kinases: How do they sense the outside?. Int. J. Mol. Sci..

[74] Okuda S. (2021). Molecular mechanisms of plant peptide binding to receptors. Peptides.

[75] Reymond P. (2021). Receptor kinases in plant responses to herbivory. Curr. Opin. Biotechnol..

[76] Sigrist C.J.A., de Castro E., Cerutti L., Cuche B.A., Hulo N., Bridge A., Bougueleret L., Xenarios I. (2013). New and continuing developments at PROSITE. Nucleic Acids Res..

[77] Finn R., Coggill P., Eberhardt R., Eddy S., Mistry J., Mitchell A., Potter S., Punta M., Qureshi M., Sangrador-Vegas A. (2016). The Pfam protein families database: Towards a more sustainable future. Nucleic Acids Res..

[78] Blum M., Chang H., Chuguransky S., Grego T., Kandasaamy S., Mitchell A., Nuka G., Paysan-Lafosse T., Qureshi M., Raj S. (2021). The InterPro protein families and domains database: 20 years on. Nucleic Acids Res..

[79] Cosgrove D. (2015). Plant expansins: Diversity and interactions with plant cell walls. Curr. Opin. Plant Biol..

[80] Lombard V., Golaconda Ramulu H., Drula E., Coutinho P., Henrissat B. (2014). The carbohydrate-active enzymes database (CAZy) in 2013. Nucleic Acids Res..

[81] Savelli B., Li Q., Webber M., Jemmat A., Robitaille A., Zamocky M., Mathé C., Dunand C. (2019). RedoxiBase: A database for ROS homeostasis regulated proteins. Redox Biol..

[82] Van der Hoorn R. (2008). Plant proteases: From phenotypes to molecular mechanisms. Annu. Rev. Plant Biol..

[83] Schaller A., Stintzi A., Rivas S., Serrano I., Chichkova N.V., Vartapetian A.B., Martınez D., Guiamet J.J., Sueldo D.J., van der Hoorn R.A.L. (2018). From structure to function—A family portrait of plant subtilases. New Phytol..

[84] Edstam M., Viitanen L., Salminen T., Edqvist J. (2011). Evolutionary history of the non-specific lipid transfer proteins. Mol. Plant.

[85] Dong X., Yi H., Han C., Nou I., Hur Y. (2016). GDSL esterase/lipase genes in *Brassica rapa* L.: Genome-wide identification and expression analysis. Mol. Genet. Genom..

[86] Hayashi S., Ishii T., Matsunaga T., Tominaga R., Kuromori T., Wada T., Shinozaki K., Hirayama T. (2008). The glycerophosphoryl diester phosphodiesterase-like proteins SHV3 and its homologs play important roles in cell wall organization. Plant Cell Physiol..

[87] Zhou K. (2019). Glycosylphosphatidylinositol-anchored proteins in *Arabidopsis* and one of their common roles in signaling transduction. Front. Plant Sci..

[88] Seifert G.J., Roberts K. (2007). The biology of arabinogalactan proteins. Annu. Rev. Plant Biol..

[89] Tavormina P., De Coninck B., Nikonorova N., De Smet I., Cammue B. (2015). The plant peptidome: An expanding repertoire of structural features and biological functions. Plant Cell.

[90] Chen Y., Ye D., Held M.A., Cannon M.C., Tui R., Saha P., Frye A.N., Mort A.J., Kieliszewski M.J. (2015). Identification of the abundant hydroxyproline-rich glycoproteins in the root walls of wild-type Arabidopsis, an *ext3* mutant line, and its phenotypic revertant. Plants.

[91] Paniagua C., Bilkova A., Jackson P., Dabravolski S., Riber W., Didi V., Houser J., Gigli-Bisceglia N., Wimmerova M., Budínská E. (2017). Dirigent proteins in plants: Modulating cell wall metabolism during abiotic and biotic stress exposure. J. Exp. Bot..

[92] Dissanayaka D., Ghahremani M., Siebers M., Wasaki J., Plaxton W. (2021). Recent insights into the metabolic adaptations of phosphorus-deprived plants. J. Exp. Bot..

[93] Sousa A.O., Assis E.T., Pirovani C.P., Alvim F.C., Costa M.G. (2014). *Phosphate-induced-1* gene from Eucalyptus (*EgPHI-1*) enhances osmotic stress tolerance in transgenic tobacco. Genet. Mol. Res..

[94] Wang T., Chen X., Zhu F., Li H., Li L., Yang Q., Chi X., Yu S., Liang X. (2013). Characterization of peanut germin-like proteins, AhGLPs in plant development and defense. PLoS ONE.

[95] Fry S.C. (2004). Primary cell wall metabolism: Tracking the careers of wall polymers in living plant cells. New Phytol..

[96] Shinohara N., Sunagawa N., Tamura S., Yokoyama R., Ueda M., Igarashi K., Nishitani K. (2017). The plant cell-wall enzyme AtXTH3 catalyses covalent cross-linking between cellulose and cello-oligosaccharide. Sci. Rep..

[97] Strohmeier M., Hrmova M., Fischer M., Harvey A., Fincher G., Pleiss J. (2009). Molecular modeling of family GH16 glycoside hydrolases: Potential roles for xyloglucan transglucosylases/hydrolases in cell wall modification in the poaceae. Protein Sci..

[98] Liu Y., Lu S., Zhang J., Liu S., Lu Y. (2007). A xyloglucan endotransglucosylase/hydrolase involves in growth of primary root and alters the deposition of cellulose in *Arabidopsis*. Planta.

[99] Minic Z., Jouanin L. (2006). Plant glycoside hydrolases involved in cell wall polysaccharide degradation. Plant Physiol. Biochem..

[100] Levy A., Erlanger M., Rosenthal M., Epel B. (2007). A plasmodesmata-associated β-1,3-glucanase in Arabidopsis. Plant J..

[101] Rhee S., Osborne E., Poindexter P., Somerville C. (2003). Microspore separation in the *quartet 3* mutants of Arabidopsis is impaired by a defect in a developmentally regulated polygalacturonase required for pollen mother cell wall degradation. Plant Physiol..

[102] Guénin S., Mareck A., Rayon C., Lamour R., Assoumou Ndong Y., Domon J., Sénéchal F., Fournet F., Jamet E., Canut H. (2011). Identification of pectin methylesterase 3 as a basic pectin methylesterase isoform involved in adventitious rooting in *Arabidopsis thaliana*. New Phytol..

[103] Minic Z. (2008). Physiological roles of plant glycoside hydrolases. Planta.

[104] Van Hengel A., Tadesse Z., Immerzeel P., Schols H., van Kammen A., De Vries S. (2001). *N*-acetylglucosamine and glucosamine-containing arabinogalactan proteins control somatic embryogenesis. Plant Physiol..

[105] Orlando M., Buchholz P., Lotti M., Pleiss J. (2021). The GH19 Engineering Database: Sequence diversity, substrate scope, and evolution in glycoside hydrolase family 19. PLoS ONE.

[106] Kesari P., Narhari Patil D., Kumar P., Tomar S., Kumar Sharma A., Kumar P. (2015). Structural and functional evolution of chitinase-like proteins from plants. Proteomics.

[107] Wan H., Wu L., Yang Y., Zhou G., Ruan Y. (2018). Evolution of sucrose metabolism: The dichotomy of invertases and beyond. Trends Plant Sci..

[108] Proels R., Hückelhoven R. (2014). Cell-wall invertases, key enzymes in the modulation of plant metabolism during defence responses. Mol. Plant Pathol..

[109] Stührwohldt N., Ehinger A., Thellmann K., Schaller A. (2020). Processing and formation of bioactive CLE40 peptide are controlled by posttranslational proline hydroxylation. Plant Physiol Biochem.

[110] Von Groll U., Berger D., Altmann T. (2002). The subtilisin-like serine protease SDD1 mediates cell-to-cell signaling during Arabidopsis stomatal development. Plant Cell.

[111] Xia Y., Suzuki H., Borevitz J., Blount J., Guo Z., Patel K., Dixon R.A., Lamb C. (2004). An extracellular aspartic protease functions in Arabidopsis disease resistance signaling. EMBO J..

[112] Wang Y., Bouchabke-Coussa O., Lebris P., Antelme S., Soulhat C., Gineau E., Dalmais M., Bendahmane A., Morin H., Mouille G. (2015). LACCASE5 is required for lignification of the *Brachypodium distachyon* culm. Plant Physiol..

[113] Pourcel L., Routaboul J.M., Kerhoas L., Caboche M., Lepiniec L., Debeaujon I. (2005). *TRANSPARENT TESTA_10_* encodes a laccase-like enzyme involved in oxidative polymerization of flavonoids in Arabidopsis seed coat. Plant Cell.

[114] Sedbrook J.C., Carroll K.L., Hung K.F., Masson P.H., Somerville C.R. (2002). The Arabidopsis *SKU5* gene encodes an extracellular glycosyl phosphatidylinositol-anchored glycoprotein involved in directional root growth. Plant Cell.

[115] Duan Y., Wang L., Li X., Wang W., Wang J., Liu X., Zhong Y., Cao N., Tong M., Ge W. (2021). Arabidopsis SKU5 Similar 11 and 12 play crucial roles in pollen tube integrity, growth and guidance. Plant J..

[116] Benedetti M., Verrascina I., Pontiggia D., Locci F., Mattei B., De Lorenzo G., Cervone F. (2018). Four Arabidopsis berberine bridge enzyme-like proteins are specific oxidases that inactivate the elicitor-active oligogalacturonides. Plant J..

[117] Jacq A., Burlat V., Jamet E. (2018). Plant cell wall proteomics as a strategy to reveal candidate proteins involved in extracellular lipid metabolism. Curr. Protein Pept. Sci..

[118] DeBono A., Yeats T., Rose J., Bird D., Reinhard Jetter R., Kunst L., Samuels L. (2009). Arabidopsis LTPG Is a glycosylphosphatidylinositol-anchored lipid transfer protein required for export of lipids to the plant surface. Plant Cell.

[119] Kim H., Lee S., Kim H., Min M., Hwang I., Suh M. (2012). Characterization of glycosylphosphatidylinositol-anchored lipid transfer protein 2 (LTPG2) and overlapping function between LTPG/LTPG1 and LTPG2 in cuticular wax export or accumulation in *Arabidopsis thaliana*. Plant Cell. Physiol..

[120] Jacq A., Pernot C., Martinez Y., Domergue F., Payré B., Jamet E., Burlat V., Pacquit V. (2017). The Arabidopsis Lipid Transfer Protein 2 (AtLTP2) is involved in cuticle-cell wall interface integrity and in etiolated hypocotyl permeability. Front. Plant Sci..

[121] Ding L., Li M., Wang W., Cao J., Wang Z., Zhui K., Yang Y., Li Y., Tan X. (2019). Advances in plant GDSL lipases: From sequences to functional mechanisms. Acta Physiol. Plant..

[122] Girard A.L., Mounet F., Lemaire-Chamley M., Gaillard C., Elmorjani K., Vivancos J., Runavot J.L., Quemener B., Petit J., Germain V. (2012). Tomato GDSL 1 is required for cutin deposition in the fruit cuticle. Plant Cell.

[123] Tsugama D., Fugino K., Liu S., Takano T. (2020). GDSL-type esterase/lipase gene, *GELP77*, is necessary for pollen dissociation and fertility in Arabidopsis. Biochem. Biophys. Res. Commun..

[124] Ursache R., De Jesus Vieira Teixeira C., Dénervaud Tendon V., Gully K., De Bellis D., Schmid-Siegert E., Andersen T., Shekhar V., Calderon S., Pradervand S. (2021). GDSL-domain proteins have key roles in suberin polymerization and degradation. Nat. Plants.

[125] Hosmani P., Kamiya T., Danku J., Naseer S., Geldner N., Guerinot M., Salt D. (2013). Dirigent domain-containing protein is part of the machinery required for formation of the lignin-based Casparian strip in the root. Proc. Natl. Acad. Sci. USA.

[126] Dunnwell J., Gibbings J., Mahmood T., Naqvi S. (2008). Germin and germin-like proteins: Evolution, structure, and function. Crit. Rev. Plant Sci..

[127] Barman A., Banerjee J. (2015). Versatility of germin-like proteins in their sequences, expressions, and functions. Funct. Integr. Genom..

[128] De Jesús-Pires C., Costa Ferreira-Neto J., Bezerra-Neto J., Kido E., de Oliveira Silva R., Pandolfi V., Wanderley-Nogueira A., Binneck E., da Costa A., Pio-Ribeiro G. (2020). Plant thaumatin-like proteins: Function, evolution and biotechnological applications. Curr. Protein Pept. Sci..

[129] Xu S., Medzihradszky K., Wang Z., Burlingame A., Chalkley R. (2016). *N*-Glycopeptide profiling in Arabidopsis inflorescence. Mol. Cell. Proteom..

[130] Tran H.T., Qian W., Hurley B.A., She Y.M., Wang D., Plaxton W.C. (2010). Biochemical and molecular characterization of AtPAP12 and AtPAP26: The predominant purple acid phosphatase isozymes secreted by phosphate-starved *Arabidopsis thaliana*. Plant Cell Environ..

[131] Seifert G. (2018). Fascinating fasciclins: A surprisingly widespread family of proteins that mediate interactions between the cell exterior and the cell surface. Int. J. Mol. Sci..

[132] Pinski A., Roujol D., Pouzet C., Bordes L., San Clemente H., Hoffmann L., Jamet E. (2021). Comparison of mass spectrometry data and bioinformatics predictions to assess the bona fide localization of proteins identified in cell wall proteomics studies. Plant Sci..

[133] Griffiths J., Crepeau M., Ralet M., Seifert G., North H. (2016). Dissecting seed mucilage adherence mediated by FEI and SOS5. Front. Plant Sci..

[134] MacMillan C., Taylor L., Bi Y., Southerton S., Evans R., Spokevicius A. (2015). The fasciclin-like arabinogalactan protein family of *Eucalyptus grandis* contains members that impact wood biology and biomechanics. New Phytol..

[135] Moussu S., Broyart C., Santos-Fernandez G., Augustin S., Wehrle S., Grossniklaus U., Santiago J. (2020). Structural basis for recognition of RALF peptides by LRX proteins during pollen tube growth. Proc. Natl. Acad. Sci. USA.

[136] Vázquez-Lobo A., Roujol D., Zuñiga-Sánchez E., Albenne C., Piñero D., de Buen A.G., Jamet E. (2012). The highly conserved spermatophyte cell wall DUF642 protein family: Phylogeny and first evidence of interaction with cell wall polysaccharides in vitro. Mol. Phylogenet. Evol..

[137] Cruz-Valderrama J., Gómez-Maqueo X., Salazar-Iribe A., Zúñiga-Sánchez E., Hernández-Barrera A., Quezada-Rodríguez E., Gamboa-deBuen A. (2019). Overview of the role of cell wall DUF642 proteins in plant development. Int. J. Mol. Sci..

[138] Jeffery C.J. (2005). Mass spectrometry and the search for moonlighting proteins. Mass Spectrom. Rev..

[139] Pinedo M., Regente M., Elizalde M., Quiroga I., Pagnussat L.A., Jorrin-Novo J., Maldonado A., de la Canal L. (2012). Extracellular sunflower proteins: Evidence on non-classical secretion of a jacalin-related lectin. Protein Pept. Lett..

[140] Regente M., Pinedo M., San Clemente H., Balliau T., Jamet E., de la Canal L. (2017). Plant extracellular vesicles are incorporated by a fungal pathogen and inhibit its growth. J. Exp. Bot..

[141] Wang J., Ding Y., Wang J., Hillmer S., Miao Y., Lo S., Wang X., Robinson D., Jiang L. (2010). EXPO, an exocyst-positive organelle distinct from multivesicular endosomes and autophagosomes, mediates cytosol to cell wall exocytosis in Arabidopsis and tobacco cells. Plant Cell.

[142] Pinedo M., de la Canal L., de Marcos Lousa C. (2021). A call ofr rigor and standardization in plant extracellular vesicle research. J. Extracell Vesicles.

[143] Rutter B., Innes R. (2017). Extracellular vesicles isolated from the leaf apoplast carry stress-response proteins. Plant Physiol..

[144] Movahed N., Garcia Cabanillas D., Wan J., Vali H., Laliberté J.F., Zheng H. (2021). Turnip Mosaic Virus components are released into the extracellular space by vesicles in infected leaves. Plant Physiol..

[145] Cai Q., Qiao L., Wang M., He B., Lin F., Palmquist J., Huang S., Jin H. (2018). Plants send small RNAs in extracellular vesicles to fungal pathogen to silence virulence genes. Science.

[146] Boevink P. (2018). Exchanging missives and missiles: The roles of extracellular vesicles in plant–pathogen interactions. J. Exp. Bot..

